# Human DNA Glycosylase NEIL1’s Interactions with Downstream Repair Proteins Is Critical for Efficient Repair of Oxidized DNA Base Damage and Enhanced Cell Survival

**DOI:** 10.3390/biom2040564

**Published:** 2012-11-15

**Authors:** Muralidhar L. Hegde, Pavana M. Hegde, Dutta Arijit, Istvan Boldogh, Sankar Mitra

**Affiliations:** 1 Department of Biochemistry and Molecular Biology, University of Texas Medical Branch (UTMB) at Galveston, Texas 77555-1079, USA; Email: mlhegde@utmb.edu (M.L.H.); padixit@utmb.edu (P.M.H.); ardutta@utmb.edu (D.A.); 2 Department of Neurology, University of Texas Medical Branch (UTMB) at Galveston, Texas 77555, USA; 3 Department of Microbiology and Immunology, University of Texas Medical Branch (UTMB) at Galveston, Texas 77555, USA; Email: sboldogh@utmb.edu (I.B.)

**Keywords:** NEIL1, DNA glycosylase, base excision repair, protein-protein interaction, reactive oxygen species, common interaction domain, disordered structure, oxidative base damage and repair

## Abstract

NEIL1 is unique among the oxidatively damaged base repair-initiating DNA glycosylases in the human genome due to its S phase-specific activation and ability to excise substrate base lesions from single-stranded DNA. We recently characterized NEIL1’s specific binding to downstream canonical repair and non-canonical accessory proteins, all of which involve NEIL1’s disordered C-terminal segment as the common interaction domain (CID). This domain is dispensable for NEIL1’s base excision and abasic (AP) lyase activities, but is required for its interactions with other repair proteins. Here, we show that truncated NEIL1 lacking the CID is markedly deficient in initiating *in vitro* repair of 5-hydroxyuracil (an oxidative deamination product of C) in a plasmid substrate compared to the wild-type NEIL1, thus suggesting a critical role of CID in the coordination of overall repair. Furthermore, while NEIL1 downregulation significantly sensitized human embryonic kidney (HEK) 293 cells to reactive oxygen species (ROS), ectopic wild-type NEIL1, but not the truncated mutant, restored resistance to ROS. These results demonstrate that cell survival and NEIL1-dependent repair of oxidative DNA base damage require interactions among repair proteins, which could be explored as a cancer therapeutic target in order to increase the efficiency of chemo/radiation treatment.

## 1. Introduction

**Figure 1:**
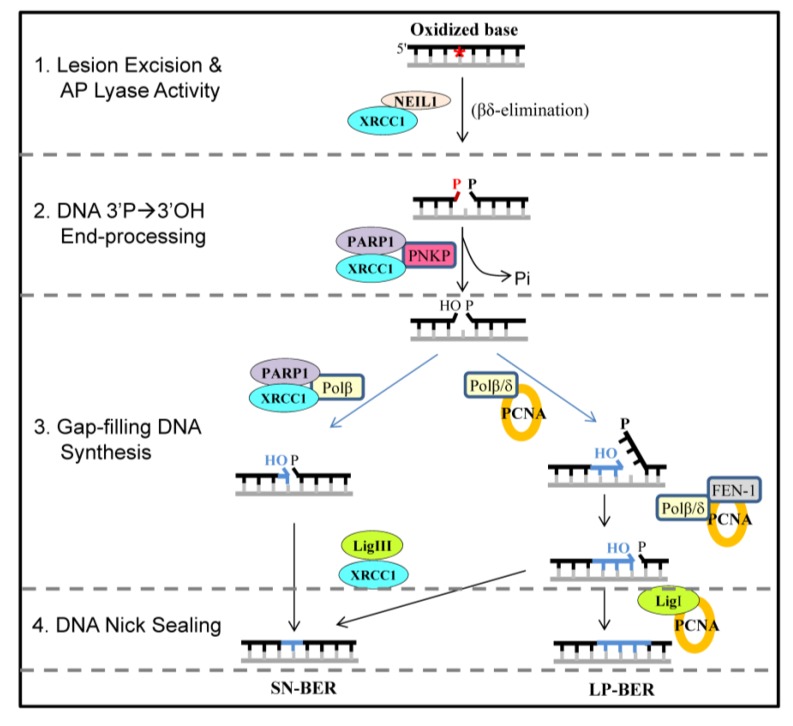
Schematic representation of NEIL1-initiated base excision repair (BER) sub-pathways in mammalian cells. Excision of the base lesion and subsequent abasic (AP) lyase activity of NEIL1 causing βδ-elimination generates a 1-nt gap at single-strand break with 3' and 5' phosphate (P) ends. The 3'P is removed by polynucleotide kinase 3' phosphatase (PNKP) in the next step to produce 3'OH which serves as primer for gap filling synthesis; this may involve incorporation of 1 nt (SN-BER) by Polβ, or of 2–8 nts (LP-BER) by Polδor Polβ in collaboration with FEN-1. Other details are given in the text.

Reactive oxygen species (ROS), generated endogenously during cellular respiration or induced after exposure to various exogenous agents/stress, inflict oxidative damage on macromolecules, including DNA, and are implicated in various human pathologies, including aging, age-related neurodegenerative diseases, arthritis and cancer [1,2,3,4,5,6,7]. ROS-induced oxidized DNA bases are repaired by the evolutionarily conserved base excision repair (BER) pathway involving four major reaction steps – excision of the base lesion followed by incision of the DNA strand by a DNA glycosylase (DG); processing of the unligatable, blocked termini at strand-break gap by an end-cleaning enzyme; gap-filling incorporation of the missing nucleotide (nt) by a DNA polymerase, and finally, nick sealing by a DNA ligase to restore genomic integrity [3,8]. Figure 1 outlines the steps in NEIL1-initiated BER.

Five oxidized base-specific DGs have been characterized in mammalian cells, and are classified in two families – OGG1 and NTH1, belonging to the Nth family, *vs*. the Nei family consisting of NEIL1, NEIL2 and NEIL3. The two families, named after their bacterial prototypes, endonuclease III (Nth) and endonuclease VIII (Nei), respectively [8,9,10,11], are distinct in their abasic (AP) lyase reaction mechanism; OGG1 and NTH1 incise the DNA strand via β-lyase activity generating 3'dRP and 5'P, while the NEIL1/2 have βδ-lyase activity, thus generating 3'P and 5'P at the strand-gap [8,12]. The 3'dRP and 3'P are then removed by AP endonuclease 1 (APE1) and polynucleotide kinase 3' phosphatase (PNKP), respectively, to generate a polymerase-ready 3'OH residue [12]. Gap filling can involve 1-nt incorporation by DNA polymerase β (Polβ) in the short-patch repair (also named single nucleotide incorporation repair, SN-BER) sub-pathway, or displacement synthesis of 2-8 nts by either Polβ or replicative DNA polymerase (Polδ) in the long-patch repair (LP-BER) sub-pathway. LP-BER requires flap endonuclease 1 (FEN-1), which removes the displaced flap oligo to allow ligation by DNA ligase IIIα (LigIIIα) or Ligase I (LigI). While SN-BER is generally believed to occur in most cells, LP-BER could occur mostly in replicating cells, where the replication enzymes are co-opted for repair [13,14]. Several accessory proteins may also play a role in BER depending on the cellular state, including the scaffold protein XRCC1 [15,16], single-strand break sensor protein PARP-1 [17], RNA-binding protein hnRNP-U [18,19], Werner helicase (WRN; [20]) and other DNA replication proteins including the sliding clamp PCNA [21], and single-strand DNA-binding replication protein A (RPA; [22]). 

NEIL1, co-discovered in our laboratory along with NEIL2 [10,11,23,24], is unique among DGs for its S phase-specific activation. Furthermore, both NEIL1 and NEIL2 excise base lesions from single-stranded DNA, unlike OGG1 and NTH1 which are active only on duplex DNA substrates [25]. NEIL1 associates with several proteins of the DNA replication machinery both *in vitro* and *in-cell,* suggesting its preferential repair role during DNA replication [20,21,22,26]. 

**Table 1:** NEIL1’s interactions with downstream canonical repair and accessory proteins involved in BER use a common interaction domain in its C-terminus. The relevant references are indicated.

Interaction partner of NEIL1	Binding region in NEIL1	Reference
Polβ	aa 312-349	[12]; present study
LigIIIα	aa 312-349	[12]; present study
XRCC1	aa 312-349	[12]; present study
FEN-1	aa 312-349	[26]
PCNA	aa 289-349	[21]
RPA	aa 312-349	[22]
hnRNP-U	aa 312-349	[18]
PARP-1	aa 312-389	unpublished observation

We have previously shown that NEIL1 directly interacts with downstream conventional repair as well as non-canonical accessory proteins (Table 1) via its CID near the C-terminus [12,18,20,21,22,26]. This region is predicted to have an intrinsically disordered conformation, based on PONDR modeling [8,27], consistent with its required deletion to obtain a crystallizable form of the protein [28]. However, the physiological significance of NEIL1’s binary interaction with most of the downstream repair proteins (including the ligases) via the CID is not understood. Here, we demonstrate the requirement of these interactions for optimum repair of oxidatively damaged bases, resulting in enhanced cell survival. 

## 2. Results and Discussion

### 2.1. The CID Is Dispensable for NEIL1’s Glycosylase Activity in vitro, but Provides a Common Interaction Region for Protein-Protein Interactions

**Figure 2:**
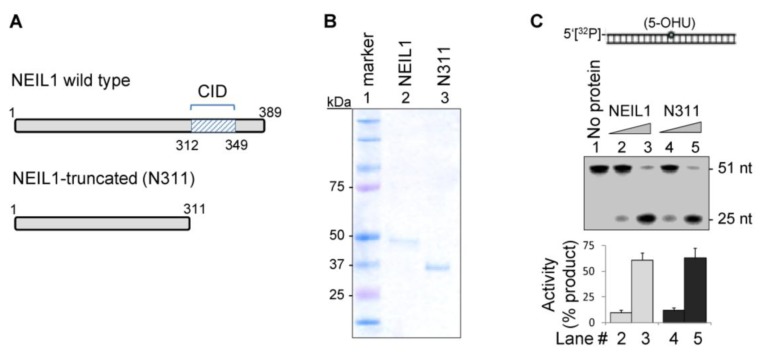
NEIL1’s common interaction domain (CID)-containing C-terminus is dispensable for DNA glycosylase activity *in vitro.* Recombinant wild-type (WT) and truncated (N311) mutant of NEIL1 (A; Coomassie-stained gel in B) show similar DNA glycosylase/AP lyase activity with a 5-OHU-containing 5'-^32^P-labeled 51-mer oligonucleotide duplex substrate to produce 25 nt oligo (C). Lanes 2 and 3: 10 and 50 fmol WT NEIL1; lanes 4 and 5: 10 and 50 fmol N311 mutant.

For several years our laboratory has focused on characterizing NEIL1’s interactions with downstream repair proteins, and identified its pairwise binding to XRCC1, Polβ, LigIIIα [12], FEN-1 [26], PCNA [21], RPA [22], WRN [20] and hnRNP-U [18]. We mapped all of these interactions to approximately 100 residues at the C-terminus of NEIL1, encompassing a minimally required 38 residue CID segment (Table 1). The DNA glycosylase/AP lyase activity of purified, recombinant wild-type (WT) *vs*. the C-terminally truncated mutant (N311, lacking the CID) were comparable with a 5'-^32^P-labeled 51-nt duplex oligo substrate containing 5-hydroxyuracil (5-OHU; Figure 2). Thus the deletion of the C-terminus has no major impact on NEIL1’s lesion excision and strand incision activities. 

**Figure 3:**
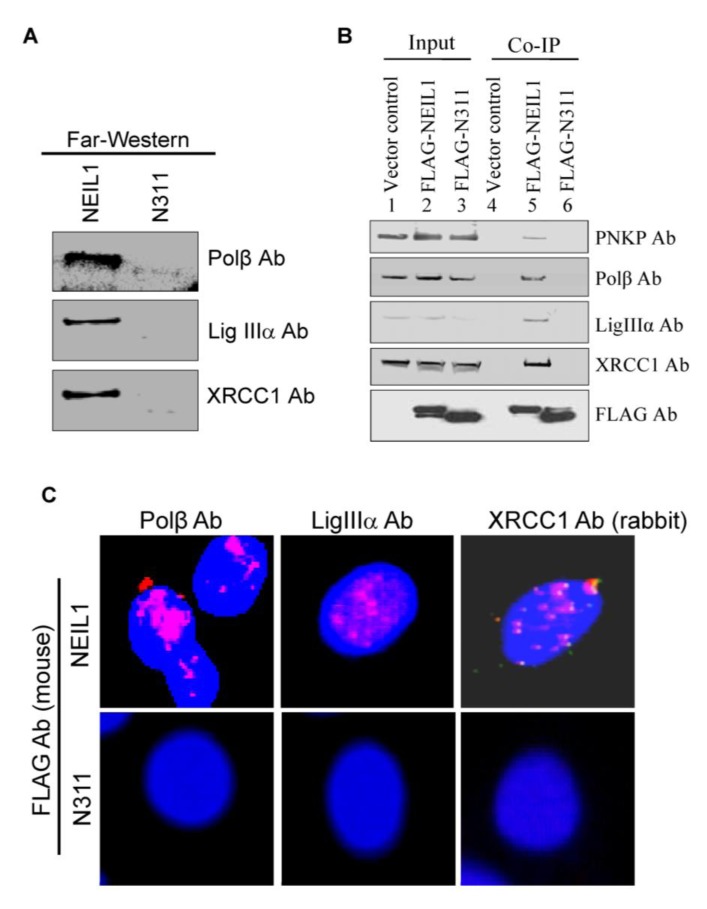
NEIL1’s C-terminus provides CID for its partner proteins. Far Western analysis with purified proteins (A), and FLAG co-IP analysis in HEK293 cell extracts expressing FLAG-WT NEIL1 or the FLAG-N311 mutant (B) show the requirement of the CID for NEIL1’s interactions with downstream SN-BER proteins. (C) PLA analysis confirms *in-cell* association of SN-BER proteins in the nucleus with ectopic WT NEIL1 but not the N311 mutant. Other details are in the Experimental Section.

Far-Western analysis showed that the CID is required for its pairwise interaction with the SN-BER proteins Polβ, LigIIIα and XRCC1 (Figure 3A). Their co-immunoprecipitation (co-IP) from FLAG- tagged WT but not truncated NEIL1-expressing HEK293 cell extracts using FLAG antibody (Ab)-beads further confirmed that *in-cell* association of NEIL1 with SN-BER proteins requires the CID (Figure 3B), consistent with our previous data [12]. The levels of ectopic NEIL1 in these cells were comparable to that of the endogenous enzyme (*data not shown*). We then performed *in situ* proximity ligation assay (PLA; Olink Biosciences) which is specific for detecting physically interacting proteins in a complex [18,28,29,30]. The association of FLAG Ab (mouse; SIGMA) *vs*. Abs (rabbit) for SN-BER proteins Polβ, LigIIIα or XRCC1 was tested in FLAG-NEIL1- or FLAG-N311 mutant-expressing cells. A significant number of nuclear foci were observed for FLAG-NEIL1 but not for the FLAG-N311 mutant, confirming NEIL1’s *in-cell* association with these proteins in HEK293 cell nuclei (Figure 3C). The PLA data thus provide independent evidence for the role of NEIL1’s CID for interactions with partner proteins, consistent with the results from co-IP and *in vitro* interaction analysis.

As already mentioned, NEIL1’s stable interaction with other repair and accessory proteins also utilizes its CID [18,20,21,22,26]. Thus this nonconserved segment, absent in Nei, might have been acquired as a terminal addition during evolution of the mammals [8,27]. It is interesting to note an analogous situation in the case of mammalian APE1, another critical component of BER, where its nonconserved N-terminal segment (65 residues), absent in the *E. coli* prototype Xth, is involved in all known interactions with partner proteins [31,32,33]. Although it is intriguing how NEIL1 or any other protein could simultaneously bind to so many proteins with high specificity via a small common peptide segment, recent studies have indicated that it is not uncommon for the mammalian hub proteins with multiple partners to have such an interaction surface, which invariably has a disordered structure [34,35]. The flexibility of the disordered domain may be critical to facilitate specific initial interactions with diverse partners [27]. These multiple interactions involving both the repair-initiating and the terminal enzyme in the pathway, along with many proteins participating in the intermediate steps, may also be important for the selection of repair sub-pathway and its efficient co-ordination [36,37]. We have previously identified specific amino acid (aa) residues in NEIL1’s CID that are involved in direct interaction with FEN-1, disruption of which significantly affects NEIL1-initiated LP-BER [26]. Furthermore, the lack of the interacting domain in NEIL1’s bacterial prototype, Nei, underscores the importance of protein-protein interactions and complexity in mammalian BER.

### 2.2. NEIL1’s CID Is Required for Efficient Repair of Oxidized DNA Bases

**Figure 4:**
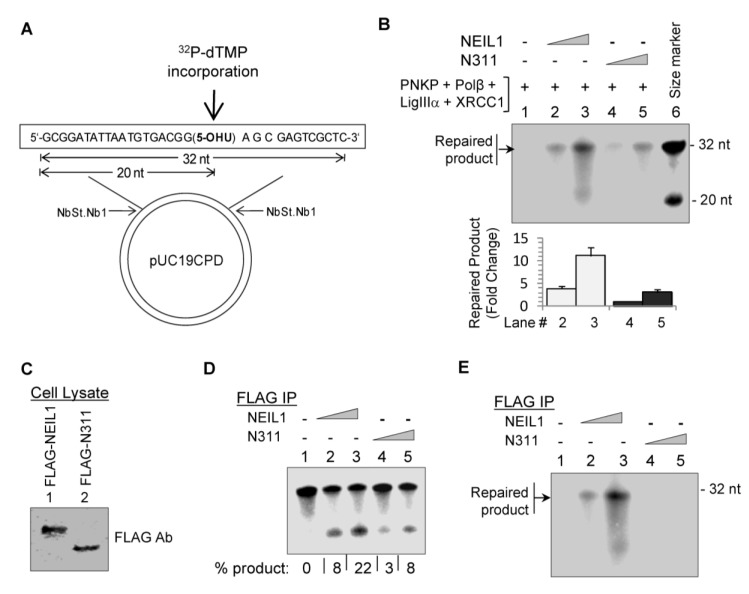
NEIL1’s CID is required for efficient repair of oxidized DNA lesions. (A) Repair of the 5-OHU-containing plasmid was monitored by the incorporation of ^32^P-dTMP and analysis of a 32 nt Nt.BstNB1 restriction repaired fragment after denaturing gel electrophoresis [18,38]. (B) *In vitro* reconstitution of NEIL1-intitiated SN-BER with purified proteins (10 and 50 fmol WT NEIL1 [lanes 2 and 3] or N311mutant [lanes 4 and 5] and 50 fmol each of PNKP, Polβ, LigIIIα and XRCC1). 5'-^32^P-labeled 32 and 20-mer oligos were used as size markers (lane 6). The histogram shows quantitation of repair. (C-E) FLAG-N311 IP, with reduced DNA glycosylase activity compared to FLAG-WT NEIL1 IP (D), was extremely deficient in overall repair (E). The DNA glycosylase activity was measured with 5-OHU-duplex oligo and complete repair with the plasmid substrate. FLAG levels in stably expressing cells were compared by Western analysis (C).

We next examined the effect of deleting NEIL1’s CID on the complete repair of 5-OHU, using either purified proteins or a FLAG IP from HEK293 cells. The repair reaction was reconstituted with a 5-OHU-containing plasmid substrate (Figure 3A), generated as described in the Experimental Section. Repair via SN-BER initiated by the N311 mutant in the presence of PNKP, Polβ, LigIIIα and XRCC1 was ~3-fold less efficient relative to WT NEIL1 (Figure 3B), as indicated by incorporation α-^32^P-TMP at the damaged base site. We then analyzed the SN-BER reaction with FLAG IPs isolated from HEK293 cell extracts stably expressing FLAG-WT NEIL1 or FLAG-N311 mutant. After confirming the comparable FLAG level in the cell extracts by Western analysis (Figure 4C), the repair protein complexes were isolated using anti-FLAG Ab-bound beads as before. The DNA glycosylase activity of FLAG-N311 IP that lacks the interacting proteins (Figure 2E; Table 1) is ~2.5-fold less than that of FLAG IP of WT NEIL1, with a 5-OHU-containing duplex oligo substrate (Figure 4D). 

We have previously shown that NEIL1’s interacting partners (e.g., FEN-1, PCNA, WRN and hnRNP-U) stimulate its glycosylase activity, which requires their binding via NEIL1’s CID [18,20,21,26]. Thus the reduced activity of the FLAG-N311 IP likely reflected the lack of its stimulation by other proteins, although the role of posttranslational modifications cannot be ruled out. Furthermore, unlike the IP of FLAG-WT NEIL1, the FLAG-N311 mutant IP was significantly deficient in complete repair of the 5-OHU-containing plasmid substrate, confirming the lack of downstream repair proteins (Figure 4E). Taken together, these results show that NEIL1’s CID, which is dispensable for its glycosylase activity, is required for its interactions with partner proteins resulting in efficient repair of oxidized bases.

### 2.3. Ectopic Wild-Type but not Truncated NEIL1 Restores Resistance to ROS Toxicity in NEIL1-Depleted Cells

**Figure 5:**
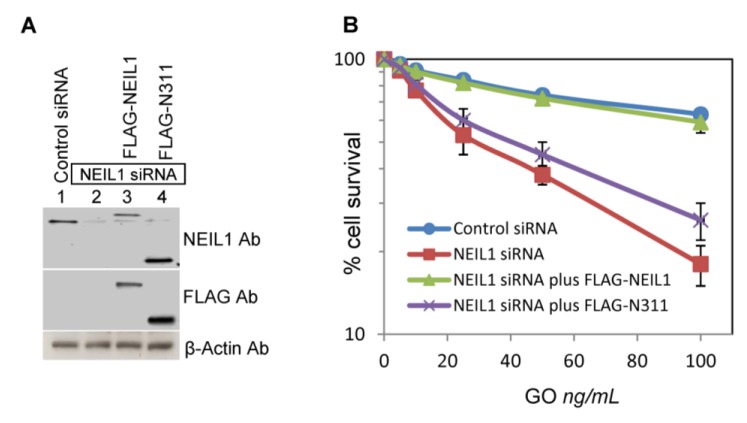
The lack of NEIL1’s CID sensitizes HEK293 cells to oxidative stress. (A) Western analysis of NEIL1 levels after its depletion by 3'-UTR specific siRNA in HEK293 cells and simultaneous expression of FLAG-WT NEIL1 or FLAG-N311 mutant polypeptide. (B) Survival of HEK293 cells transfected with control siRNA or siRNA for NEIL1, and simultaneous co-transfection of FLAG-WT NEIL1 or FLAG-N311 mutant expression plasmids. The details are in the Experimental Section.

The requirement of NEIL1’s interaction with other repair proteins for optimal repair predicts that the CID-lacking mutant would not be able to reverse the ROS sensitivity of NEIL1-deficient cells. We tested this with clonogenic survival assays for NEIL1-depleted HEK293 cells after glucose oxidase (GO) treatment (which generates H_2_O_2_), and examined the effect of ectopic expression of WT NEIL1 *vs*. the N311 mutant. Endogenous NEIL1 was depleted (>80%;) after transfection with 3'-UTR-specific siRNA (Experimental Section) that allowed ectopic expression of NEIL1’s coding sequence (Figure 5A). NEIL1 deficiency significantly increased cells’ sensitivity to GO, consistent with our previous observation [18]. However, ectopic expression of WT NEIL1, but not of its N311 mutant, protected endogenous NEIL1-depleted cells from ROS sensitivity. These results support our conclusion that NEIL1’s interactions with downstream repair and accessory proteins via its CID are required for protection of cells after oxidative stress.

## 3. Experimental Section

### 3.1. Expression and Purification of Recombinant Proteins

Recombinant WT NEIL1, PNKP, Polβ, LigIIIα and XRCC1 were purified to homogeneity from *E. coli-*bearing their expression plasmids, as described previously [10,12,38]. Truncated NEIL1 (N311) clone, an endoproteinase Asp-N limited-digestion product of NEIL1 [20], was generated by introducing stop codons after the 311^th^ aa position in a NEIL1-expression plasmid (pET22b; [10]) using Quick Change site-directed mutagenesis (Stratagene). After confirming the sequence, the untagged N311 was expressed in *E. coli* BL21-RIPL cells in LB media in a 16˚C shaker overnight and purified to homogeneity as before [10]. The purity of the NEIL1 and N311 preparations was confirmed by SDS-PAGE analysis (Figure 2B). 

### 3.2. Generation of FLAG-NEIL1 and FLAG-N311 Expression Plasmids and Their Stable Expression in HEK293 Cells

A mammalian expression plasmid (modified pcDNA-FLAG) for FLAG-tagged WT NEIL1 was described previously [18,21]. To generate the FLAG-N311 mutant expression plasmid, cDNA corresponding to this sequence was PCR-amplified from a NEIL1 expression plasmid using EcoR1/BamH1 site-containing primers and cloned in a pcDNA-FLAG plasmid. Stable transfectants of FLAG-NEIL1 and FLAG-N311 in HEK293 cells were generated by transfecting the cells with the respective plasmids and selecting clones with resistance to zeocin (100 µg/mL). The surviving clones were expanded, and after analysis of FLAG expression the clones with low FLAG level (comparable to the endogenous NEIL1) were used in this study. 

### 3.3. DNA Substrates for Repair Assay

The oligonucleotide and plasmid substrates containing the base lesion 5-OHU used in NEIL1’s glycosylase and complete repair assays, respectively, were described earlier [7,18,38]. To produce radio-labeled substrates, the single-stranded 5-OHU-containing oligo was labeled at the 5′-termini with [γ-^32^P]-ATP using T_4_-PNK (New England Biolabs) before annealing. The labeled substrates were separated from unincorporated radioactivity by chromatography on Sephadex G25. 

### 3.4. Cell Culture and Co-Immunoprecipitation

HEK293 cells were cultured in Dulbecco’s modified Eagle’s medium (DMEM) supplemented with 10% fetal bovine serum, 100 units/mL of penicillin, 100 μg/mL streptomycin and 2 mM glutamine, at 37 °C and 5% CO_2_. For co-IP analysis, log-phase cells stably expressing empty FLAG, NEIL1-FLAG, or N311-FLAG plasmids were lysed, digested with 500 units/ml benzonase (Novagen) at 37 °C for 30 min, and cleared by centrifugation. The supernatants were then immunoprecipitated for 3 h at 4^0^C with anti-FLAG Ab cross-linked to agarose beads (SIGMA). After collecting the beads by centrifugation and washing three times with cold Tris-buffered saline, the FLAG immunocomplex was eluted from the beads by adding SDS loading buffer for Western analysis. For repair assays with IPs, the beads were incubated with substrate with constant shaking [18]. 

### 3.5. Far Western Analysis

Recombinant WT NEIL1 and N311 mutant (40 pmol) were transferred to a nitrocellulose membrane after SDS-PAGE (12%), denatured *in situ* with 6M guanidine-HCl and then renatured by sequential incubation with serially diluted guanidine-HCl in PBS + 1 mM DTT [22], before incubating the membrane with Polβ, LigIIIα or XRCC1 (10 pmol/mL) in PBS supplemented with 0.5% nonfat dried milk, 0.05% Tween 20, 10 mM trimethylamine N-oxide (TMAO) and 1mM DTT for 3h, followed by immunoblotting with appropriate Abs.

### 3.6. In Situ Proximity Ligation Assay (PLA)

HEK293 cells stably expressing FLAG-WT NEIL1 or FLAG-N311 mutant were cultured overnight in an 8-well chamber slide, fixed with 4% paraformaldehyde, then permeabilized with 0.2% Tween 20, followed by incubation with a primary Ab for FLAG [mouse; SIGMA] and rabbit Abs for Polβ (a gift from Dr. S.H. Wilson, NIEHS, NC), LigIIIα (Bethyl Laboratories) or XRCC1 (Santa Cruz). PLA assays were performed using the Duolink PLA kit from OLink Bioscience (Cat# LNK-92101-KI01; Uppsala, Sweden) per the manufacturer’s instructions. The nuclei were counterstained with DAPI, and the PLA signals were visualized in a fluorescence microscope (NIKON Ti system) at 193x magnification. In this analysis, two proteins are immunostained with distinct species-speciﬁc secondary Abs that are linked to complementary oligonucleotides. When two different Ab molecules bind in close proximity (<40 nm), the linked DNA can be linearly ampliﬁed via a rolling circle mechanism and visualized as distinct foci with a ﬂuorescent probe. 

### 3.7. DNA Glycosylase/AP Lyase Assay of NEIL1

The base lesion excision and strand incision activity of WT NEIL1 or N311 mutant was analyzed after incubation of 5' ^32^P-labeled, 5-OHU-containing duplex oligo substrate at 37˚C for 15 min in a 10 μl reaction mixture containing 40 mM HEPES-KOH, pH 7.5, 50 mM KCl, 1 mM MgCl_2_, 100 μg/ml bovine serum albumin and 5% glycerol. The reaction was stopped with the formamide dye mix (80% formamide, 20 mM NaOH, 20 mM EDTA, 0.05% bromophenol blue and 0.05% xylene cyanol) and the products were analyzed in a PhosphorImager using Image Quant software after separation by denaturing gel electrophoresis [18,26]. 

### 3.8. Complete Repair Assay

Repair of 5-OHU-containing plasmid substrate initiated with WT-NEIL1 or the N311 mutant was analyzed using reconstituted *in vitro* system containing recombinant proteins or FLAG IP as described previously [7,18]. Briefly, the repair reaction (20μL) containing 50 fmol each of PNKP, Polβ, LigIIIα and XRCC1 along with 10 and 50 fmol WT NEIL1 or N311mutant together with 1 mmol ATP, 10 μmol of [α-^32^P]-dTTP, and unlabeled dNTPs (25 mmol) was incubated for 30 min at 37 °C. For repair using the FLAG IP, recombinant proteins were replaced with FLAG-Ab bead eluates after co-IP of HEK293 cell extracts with comparable FLAG levels The products were analyzed in a PhosphorImager after separation in a denaturing gel as before [26].

### 3.9. Cell Survival Assay

Log-phase HEK293 cultures were transfected with NEIL1 siRNA (80 nM; targeting the 3'UTR region of the NEIL1 gene; sense sequence, 5'CCGUGAUGAUGUUUGUUUAUU3'; antisense sequence, 5'UAAACAAACAUCAUCACGGUU3', SIGMA; [18]) or scrambled control siRNA. NEIL1’s depletion was confirmed by immunoblotting the cell extracts at 48 h after transfection. Separately, cells were co-transfected with NEIL1 siRNA plus FLAG-WT NEIL1 or FLAG-N311 mutant expression plasmid. After 48 h, the cells were treated with GO (0 to 100 ng/mL; in triplicate) for 15 min, then trypsinized and transferred to 60-mm dishes (400 cells/dish). Cells were allowed to grow in fresh medium for 8 days; the colonies were counted after staining with crystal violet to calculate the surviving fraction [18,39].

## 4. Conclusions

BER is essential for survival of aerobic organisms in order to repair both endogenously produced and exogenously inflicted genomic damage, including ROS-induced, cytotoxic and mutagenic oxidized DNA bases [40]. Defects in BER and consequent accumulation of oxidative genomic damage have been associated with cancer susceptibility and neurodegeneration [2,3,6]. As already mentioned, while *in vitro* reconstitution of complete BER requires only a few proteins [12,38], several recent studies by us and others have documented the involvement of various noncanonical accessory proteins that physically and/or functionally interact with one or more conventional BER proteins [17,18,19,20,21,22]. Thus, multiple protein-protein interactions characterized among BER proteins not only involve canonical BER proteins (including the first and last protein in the pathway [12,38]), but also several accessory proteins and proteins involved in other DNA transaction pathways. These multiple interactions could thus play one or many of the following roles: (i) stabilizing the interacting proteins; (ii) recruiting specific partner(s) to the damaged/intermediate repair site; (iii) protecting the toxic DNA repair intermediates; (iv) controlling repair sub-pathway selection; (v) modulating repair activity; and (vi) coordinating sequential steps in repair. Many of these interactions could be affected by posttranslational modifications in the interacting partners [3,36]. In this study, we demonstrated that interactions of the major BER-initiating enzyme NEIL1 with other repair and accessory proteins are important for efficient repair of oxidized DNA bases and for cellular survival after oxidative stress. 

As cellular sensitivity to agents that inflict genome damage is dependent on the DNA repair competence of the cells, various ways of targeting the BER process are being explored for sensitizing cancer cells as an adjunct modality to radiation or radiomimetic drug therapy [41,42,43,44]. Our studies showing the critical role of interactions among BER proteins suggest that these protein-protein interactions, typically involving a common binding interface, as in NEIL1, should be explored as a target to enhance chemo/radiation sensitivity of cancer cells. These potential new targets may be particularly important when the surviving cancer cells develop resistance to conventional DNA repair inhibitors. 
